# Analysis of the on-ship transmission of the COVID-19 mass outbreak on the Republic of Korea Navy amphibious warfare ship

**DOI:** 10.4178/epih.e2022065

**Published:** 2022-08-11

**Authors:** Soo Hyeon Cho, Young-Man Kim, Gyeongyong Seong, Sunkyun Park, Seoncheol Park, Sang-Eun Lee, Young Joon Park

**Affiliations:** 1 Central Disease Control Headquarters, Korea Disease Control and Prevention Agency (KDCA), Cheongju, Korea; 2 Armed Forces Epidemiological Investigation Center, Seongnam, Korea; 3 Office of the Surgeon General, Republic of Korea Navy Headquarters, Gyeryong, Korea; 4 Department of Information Statistics, Chungbuk National University, Cheongju, Korea

**Keywords:** COVID-19, Outbreaks, Epidemiology, Military personnel, Ships

## Abstract

**OBJECTIVES:**

This investigation was conducted to determine the size and pattern, source, and transmission route of the coronavirus disease 2019 (COVID-19) outbreak on the Republic of Korea Navy (ROKN) amphibious warfare ship.

**METHODS:**

We investigated the characteristics of all crew members and tracked the medical records of the confirmed cases. Fourteen essential ship operation personnel were interviewed. The study design was a retrospective cohort study, and the incidence rate ratio was through a statistical program.

**RESULTS:**

The COVID-19 incidence on the ROKN amphibious warfare ship was 44.7% (38/85). It was estimated that the main propagation route started from the 1st floor worker, which spread to the same floor, and then to other floors. In the case of the working area, the incidence rate of crew members below the 1st floor without ventilation was higher than those on the 2nd or higher floors with natural ventilation.

**CONCLUSIONS:**

This case is the first case of a COVID-19 outbreak on the ROKN amphibious warfare ship, and it is estimated that the incidence rate is high because of the closed and dense environment. To prevent the spread of various respiratory diseases including COVID-19, unified mitigation such as vaccination, observing personal quarantine rules, periodic ventilation, preemptive testing, and blocking transmission through prompt contact management is necessary.

## GRAPHICAL ABSTRACT

[Fig f2-epih-44-e2022065]

## INTRODUCTION

Since the first pneumonia outbreak of unknown cause in Wuhan City, Hubei, China, in December 2019, coronavirus disease 2019 (COVID-19) has spread across the world. The main route of transmission of COVID-19 is known to be through respiratory droplets, while contact transmission is also possible via the media carrying the droplet containing the virus (which can survive from four hours up to several days) [1]. Airborne transmission has also been recently reported to have occurred in a closed, restricted area [2]. Clinical symptoms vary from being asymptomatic to having severe symptoms. The main symptoms include fever of 37.5°C or higher, sore throat, cough, respiratory distress, chills, muscle pain, headache, and loss of smell or taste. Since the declaration of the COVID-19 pandemic in January 2020, outbreak cases have been reported worldwide with respect to various transmission routes and places including education facilities, religious facilities, and multi-use facilities such as public baths and restaurants, hospitals, and business establishments [3,4]. In the spectrum of COVID-19 outbreak cases, those concerning ships include the case of a cruise ship of D company in Japan, where 700 people on board were infected in mid-February 2020, and subsequent cases of a cruise ship of G company in the Unite States and a cargo ship in Brazil [5-7]. Of note are the COVID-19 outbreak cases on warfare ships including aircraft carriers [8,9] that led to the suggestion of the risk of on-ship transmission and infection of COVID-19. In Korea, the cumulative number of confirmed cases was 16,583,220 as of 00:00, April 20, 2022, from the first confirmed case of COVID-19 in January 20, 2020 [10]. Various outbreak cases have occurred, but mass transmission has been effectively controlled through active adherence of the public to policies such as social distancing and mask-wearing as well as the focused management, total inspection, and preemptive testing of risk groups in the community as conducted by the disease prevention authorities and local governments. In particular, as the military personnel in communal living are known to be susceptible to various infectious diseases [11], the relevant disinfection measures were reinforced by the Ministry of Defense in consideration of the characteristics of military organizations. However, since December 2020, the variant of concern originating from the United Kingdom, the Republic of South Africa, and Brazil and the variant of interest originating from the United States have been identified in Korea; because the virus propagation, transmission, and variant formation are predicted to occur continuously [12], the investigation and analysis of outbreak cases are deemed highly important.

Since the recognition of an outbreak case on the Republic of Korea Navy (ROKN) amphibious warfare ship in April 23, 2021, the Korea Disease Control and Prevention Agency (KDCA) and the Ministry of Defense have organized the joint COVID-19 Response Team (CRT) for rapid epidemiological investigation and prevention of transmission, while the case presented in this study is the first COVID-19 outbreak case on an ROKN amphibious warfare ship. The scale and pattern of the COVID-19 outbreak and epidemiological features such as source of infection and transmission route are reported in this study.

## MATERIALS AND METHODS

### Outbreak recognition

The present investigation was conducted upon recognition of the COVID-19 outbreak on April 23, 2021, with the first confirmed case on April 22, 2021, and 32 subsequent confirmed cases identified during the total inspection of all 84 crew members of the ROKN amphibious warfare ship, which had departed from the Jinhae Navy Base, located in Changwon-si, Gyeongsangnam-do, Korea, on April 20, 2021, and had anchored at the Pyeongtaek Navy Base, in Pyeongtaek-si, Gyeonggi-do, Korea, on April 23, 2021.

### Case definition and epidemiologic investigations

A confirmed case is defined as ‘a case where the severe acute respiratory syndrome coronavirus 2 viral gene (SARS-CoV-2/E, RdRp) was detected in the reverse transcription polymerase chain reaction (RT-PCR) of a sample collected from the upper airway, based on the diagnostic criteria and irrespective of the clinical features ‘A case where the severe acute respiratory syndrome coronavirus 2 viral gene (E, RdRp) was detected in the reverse transcription polymerase chain reaction (RT-PCR) of a sample collected from the upper airway, based on the diagnostic criteria and irrespective of the clinical features’ among the cases of confirmed exposure during the period of investigation (April 18 to 22) on the ship [13]. The tests of crew members on board were performed by a secondary health care center in Pyeongtaek-si in the case of the index case, and by the Armed Forces Medical Research Institute for the remaining crew members after sample collection on the field. For sample analysis, a selected set was examined by the Armed Forces Medical Research Institute. On April 23, the day the COVID-19 outbreak on the ship was reported, the joint CRT of the KDCA and Ministry of Defense performed an on-site epidemiological investigation and response measures to identify the outbreak pattern, source of infection, and transmission route on the ship and to prevent further transmission and control the infection. According to the COVID-19 response guidelines, all confirmed individuals should be quarantined and treated in principle [13]. However, considering the characteristics of an amphibious warfare ship in the case of an emergency, a minimum number of 14 crew members working as operators were quarantined at the cohort on the ship. Hence, upon a field visit, the investigators wore personal protective gear and led the interviews with the cohort-quarantined operators. A specific questionnaire was not used, but the practical opinions regarding the way of operation on the ship and lifestyle were collected. For the medical record, the epidemiological investigator of the Armed Forces Epidemiological Investigation Center reviewed the private medical records used in the primary epidemiological investigation of confirmed cases to identify smoking status and underlying diseases, and the epidemiological investigator of the naval force collected data on demographics such as age and sex, and living and working areas of all crew members. In addition, to identify the route of transmission, the contents of the drug utilization review (DUR) by the Health Insurance Review and Assessment Service were reviewed for the confirmed cases. As such, the joint CRT conducted a retrospective cohort investigation of all crew members (n= 85) in terms of the data of the history of ship activity, field visit, interview, and medical records.

### Statistical analysis

The R version 4.1.2 (R Core Team, Vienna, Austria), and R package (especially, the rateratio function expressed by ‘fmsb’) were used for statistical analysis, at a significance level of alpha= 0.05 (95% confidence interval, CI). Through these programs, the variables predicted to influence the COVID-19 infection and the subjects’ general characteristics were assessed by estimating the crude relative risk and the adjusted relative risk after the adjustment of the effects of other variables. Such comparison of the incidence rate ratio (IRR) according to demographic characteristics was performed in reference to the equations reported in previous studies [14-17]. A 2× 2 contingency table was drawn for COVID-19 infection and other variables, and the respective analysis was performed with age, sex, rank, and working area as the selected variables. In the case of adjusted rate ratio, the Mantel–Haenszel method was used as an additional stratification method to adjust the effects of other variables. The method involves the stratification and specification of the data of the contingency table by category in the estimation of the adjusted influence of variables, where the weighted mean of rate ratio on each stratum is used to present the adjusted rate ratio free from the effects of other variables. For the variable selection criteria in the evaluation of the relative risk, the categories were reorganized in consideration of the characteristics of the military base and the physical structure of the ship, taking into account the relatively small sample size (n= 85). Age was divided into two groups (19-24 and 25-49 years) considering the characteristics of the military rank. Working area was divided into the area without ventilation (basement to 1st floor) and the area with natural ventilation (2nd to 3rd floors)

### Ethics statement

The study was approved by Republic of Korea Armed Forces Medical Command Institutional Review Board (IRB No. AFMC202202-HR-008-02).

## RESULTS

**Figure 1.**
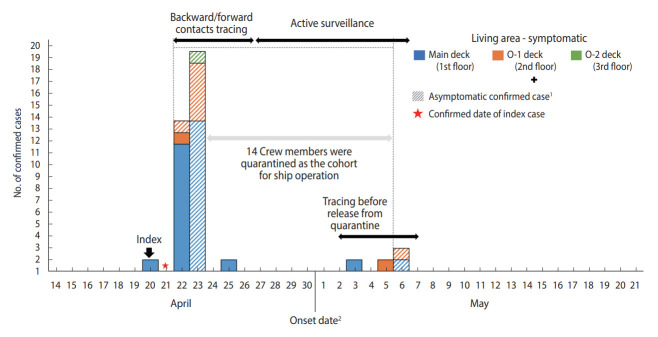
Epidemic curve of coronavirus disease 2019 confirmed cases in Republic of Korea Navy amphibious warfare ship. ^1^Shaded marks indicate asymptomatic confirmed cases. ^2^If the case is asymptomatic, it is treated as the confirmed date.

Across all 85 crew members of the ROKN amphibious warfare ship, the incidence was 44.7% with 38 confirmed cases and 47 non-confirmed cases. The report of the Armed Forces Medical Research Institute showed that no variant of concern was detected in the S protein analysis of three confirmed cases. The index case was a male patient in his 30s ranking under the Chief Petty Officer (CPO). At approximately midnight on April 21, 2021, he was informed of the incidence of numerous confirmed patients from the daycare center he had visited with his child on April 16 and immediately quarantined himself on the ship. At around 10 a.m. on April 22, as soon as the ship entered the port in Pyeongtaek-si, he was tested at the screening clinic at a local hospital and was confirmed positive at approximately 5 p.m. on the same day. Following the diagnosis of the index patient, total inspection of all crew members identified 32 confirmed patients by April 23. One additional confirmed case was identified on April 25 from a symptomatic patient, and four additional confirmed cases were identified before the crew’s release from quarantine in May (Figure 1). For the index patient, the first epidemiological investigation reported that he had been asymptomatic, but the DUR investigation found that he had a history of visiting a pharmacy and had had a cough for three weeks. However, as there had been no confirmed case on the ship during that period and no traced contact with epidemiological association and no confirmed case in the family of the index patient, the onset date of symptoms was conjectured as April 20 to set the period of investigation, and based on the recent history of a pharmacy visit and cough of three weeks duration the symptoms were determined to be irrelevant to COVID-19 infection. Thus, by presuming the index patient as the source, the ship was estimated to have been exposed to the COVID-19 virus for a minimum of five days between April 18 and April 22, the date of confirmation of the index case. While the index patient was stationed in an independent working area on the 1st floor (main deck), the structure of the ship allowed contact with other crew members at various areas on the ship such as lavatories and shared facilities. In particular, as the index patient had been in charge of supplies, close contact with many unspecified persons was possible. In addition, he had been on duty on April 18, the date estimated to be approximately two days prior to the onset of symptoms, indicating an increased probability of contact with other crew members through leading the roll calls and having meals with others on duty.

The demographic characteristics of the crew members on the ship are presented in Table 1. The percentage of those with confirmed infections was 48.1% among males (n = 77) and 12.5% among females (n= 8, one confirmed case). For age, the highest incidence was seen for those in their 40s (50.0%), but due to the characteristics of the military organization, a high incidence (21/38, 55.3%) was seen for those aged 19-24 years across all confirmed patients. For rank, the incidence was 46.7% for under CPOs and 58.5% for Senior & Master CPOs. while the Officers all tested negative. For working area, the members working on the 1st floor (main deck) showed the highest incidence at 52.5%, followed by those working on the basement (2nd deck, 42.9%) and those on the 2nd floor (O-1 deck, 33.3%); while none of those working on the 3rd floor (O-2 deck) tested positive. Lastly, for living area, the members living on the 1st and 2nd floors showed high rates of incidence at 50.9% and 56.3%, respectively, while one out of the 14 members living on the 3rd floor was confirmed to have an infection (7.1%). For clinical symptoms across confirmed patients, 57.9% (n= 22) were asymptomatic and 42.1% (n= 16) were symptomatic, the chief complaint of the latter being cough (n= 12) and headache and chills (n= 7). For demographics, most confirmed patients (97.4%) were non-smokers.

The risk of infection in relation to demographic characteristics was compared based on age, sex, rank, and working area as shown in Table 2. For the crude rate ratio without controlling other variables, the risk of infection was higher in females than in males (6.48; 95% CI, 0.89 to 7.19) and in under CPOs than in Senior or Master CPOs or Officers (1.31; 95% CI, 0.64 to 2.70), although no statistical significance was observed. The risk of infection according to working area was significantly higher in the basement and 1st floor than in the 2nd or 3rd floors (3.98; 95% CI, 1.41 to 11.23). For the adjusted rate ratio, the correlations of age, rank, and sex variables were analyzed in consideration of the unique characteristics of military organizations. Using the Cramer’s V statistic, the correlations of categorical variables were analyzed, and the highest correlation coefficient was found between age and rank (0.666). Thus, the age variable with high correlations with other variables but the lowest correlation with the confirmation of infection was excluded. The rate of infection was higher in females than in males for the adjusted rate ratio although no statistical significance was observed (4.52; 95% CI, 0.65 to 31.26). The risk of infection was lower in under CPOs than in Senior or Master CPOs or Officers although no statistical significance was observed (0.91; 95% CI, 0.44 to 1.89). As with the crude rate ratio, the rate of infection according to working area was higher in the basement and 1st floor than in the 2nd or 3rd floors, likewise with statistical significance (3.20; 95% CI, 1.14 to 8.99).

In the field investigation and interviews with crew members, it was found that the crew members generally had meals in the galley where the rationing was divided between the basement and the 1st floor, while an acryl plate was installed on the tables to maintain maximum distances among the people sitting along a single direction to minimize contact, but due to the physical restriction of the space inside the ship, distancing above 1 m was difficult. On the 1st floor, as in the basement floors, natural ventilation was difficult and the mechanical ventilation system was manually run only if a need arose. The living area (berth and galley) of Senior and Master CPOs, infirmary, and the Officer’s mess and meeting rooms were located on the 2nd floor. In contrast to the areas from the 1st floor and below, natural ventilation was possible, and distancing was easier with acryl plates installed in meeting rooms to prevent droplet transmission. There was a wardroom on the 3rd floor, and as with the 2nd floor, natural ventilation was possible. While the crew members on the ship worked in three shifts for ship maintenance, operation, and cooking, the living areas (including the berth, galley, showers, and lavatories) were strictly separated between those of the under CPOs and those of the Senior and Master CPOs and Officers. For personal arrangements prior to departure, the seamen were allowed to leave for a short while, and upper Petty Officer 1st class were also found to have had personal time to pack and do other things out of the ship. In the meantime, however, the distancing within military bases had been increased a step higher than that applied to the general public (i.e., operating at Step 2) to prevent COVID-19 outbreak. In the navy base, likewise, unnecessary leaves were prevented to minimize contact with external personnel, and upon return from leave, a PCR test was performed mandatorily. Even upon a negative PCR result, the respective individual was excluded from work for 14 days and distancing was maintained via cohort-quarantine of the returnees.

## DISCUSSION

This study was conducted to determine the scale and pattern and the source of infection and transmission route related to the COVID-19 outbreak on an ROKN amphibious warfare ship. The overall incidence on the ship was 44.7%, with no transmission to the community as there had been no other individuals on board other than the crew members, unlike cargo ships or cruise ships for tourism that had been previously investigated.

The transmission route was presumed to originate from the index patient, who had been the source with the earliest onset of symptoms as well as the first confirmed patient. In fact, most confirmed patients were crew members of the rank below under CPOs as with the index patient, while no confirmed patient was found among the Officers who had relatively little contact with the index patient. In the living area on the 3rd floor, where the Officers mostly dwelled, one confirmed case occurred; a female crew member of the rank below the under CPOs using the female lodging and who is suspected to have contracted the infection from her working area on the 1st floor. Nevertheless, no history of personal contact was found, and in identifying the transmission route, the potential contact of the index patient with other rank members during the period of transmission due to the duty or general supply work should be taken into account. Based on the structure of the ship where the working and living areas overlap, multiple simultaneous transmission could have occurred between the index patient working and living on the 1st floor and the crew members on the 2nd and 3rd floors. For the transmission route, no other crew member with an earlier onset of symptoms than the index patient was found and considering the characteristics of military organization with restricted coming and going of external personnel, the present outbreak case is presumed to have been caused by the infection of other crew members from the index patient through an unknown route of infection. Analyzing the history of visiting a health care center or pharmacy of all crew members including the index patient, showed no specific case of medical care or purchase related to COVID-19-like symptoms, and no epidemiological association was found for the contact with a confirmed patient among the general public other than with the index patient. Despite the possibility that the index patient had contact with a confirmed patient at the daycare center of his child, no family member of his had been confirmed positive for infection, thus, the route of infection could not be identified. The transmission route remains unknown for the index patient, and a confirmed patient on the ship preceding the index patient could not be identified.

Comparisons may be made on the scale of outbreak with the large-scale on-ship outbreak cases: the D company ship with an incidence of 19.2% across all crew members and people on board, and the G company ship with an incidence of 3.4% across all crew members and people on board [5-7]. Overseas warfare ship outbreaks include the case of an aircraft carrier ship with an incidence ≥ 25% [9] and the case of a warfare ship with an incidence of approximately 6.3% upon the detection of the Delta variant, a variant of concern, after the vaccination of 98% of crew members [8]. While these cases may differ in the type of virus at the time of outbreak and vaccination, no variant of concern had been detected in the present case. The type of virus is presumed to have no notable factors despite the lack of genotyping, considering that most confirmed patients at the time of outbreak at the end of April 2021 had been GH clade [12]. Domestic cases include the outbreak with an incidence of approximately 90.4% on the ship that had been on an overseas dispatch duty in July 2021, without vaccination of the crew members [18]. In this case, the Delta variant had been detected. While the virus in the present case is not a variant of concern with a relatively high level of transmission [19], compared to similar domestic and overseas outbreak cases, the incidence may be interpreted as relatively high. The cause of such a high rate of incidence may be as follows: first, the ship has been in service for about 30 years, so internal ventilation below the 1st floor is limited and at the same time vulnerable to the spread of droplets. Natural ventilation was not possible in certain places in the ship, and it was not appropriate to comply with the ventilation guidelines [13] of the KCDA because it was difficult to periodically operate the mechanical ventilation system. Therefore, it was estimated that staying or coming into contact with the living and activity space below the 1st floor where natural ventilation was not possible, was a major risk factor for group infection. As a result of the analysis of this study, the risk of transmission was lower in the 2nd floor or higher than that of the 1st or lower floors because natural ventilation was possible, and it was confirmed that the difference was statistically significant through univariate and multivariate analysis. Therefore, in areas where natural ventilation is not possible, air-conditioning facilities should be operated frequently to provide periodic ventilation. Second, it is possible to consider the use of a common restaurant by on-call workers, which could be expected as a risk factor for inter-class transmission in this case. According to the results of existing studies that a restaurant on the ship, where masks are not allowed, can be a place for the spread of COVID-19 [20], it is determined that maintaining enhanced distance while eating is important [21,22], so the distribution time is shortened to minimize contact. Meals should be carried out sequentially, and it may be necessary to prohibit conversation between meals. Third, considering that more than half of the confirmed cases on the amphibious ship were asymptomatic, the military should conduct symptom monitoring for those returning from leave, keeping in mind the maximum incubation period and the fact that known asymptomatic infections can spread [23]. It is necessary to identify symptomatic persons before boarding and departure, and to conduct a preemptive examination of all crew members before departure, considering the situation where PCR testing on board is impossible. In addition to observing preventive measures such as wearing a mask, it is required to minimize additional infections by focusing on blocking the spread of transmission, such as immediately isolating living spaces and promptly managing contacts when a person with symptoms is found after departure. Lastly, it should be considered that this cluster outbreak occurred before the introduction of COVID-19 vaccination. Considering the recent research results [8] that the incidence of COVID-19 is reduced when most crew members are vaccinated while complying with quarantine rules in a similar environment on board, it is necessary to complete vaccinations before departure.

On the other hand, this study has the following limitations. First, due to the nature of military security, it was impossible to grasp the movement patterns of crew members using CCTV in the ship, there was a limit to securing objectivity by relying only on statements, and it was also impossible to secure drawings and designs to accurately explain the structure of the ship. Second, the characteristics of the air flow in the ship, including the communal dining room, could not be grasped because the aerodynamics experiment could not be conducted due to the restrictions on the access of outsiders. Finally, at the time of the outbreak of this group case, vaccination of crew members was not carried out, so it was not possible to analyze the relationship with vaccination history and the effect of vaccination.

This investigation analyzed the status of the first COVID-19 cluster outbreak that occurred in the ROKN amphibious warfare ship, and identified the size and pattern of the cluster outbreak, and propagation route. The infection started from a crew member whose source of infection is unknown, and it can be expected that there was a simultaneous transmission due to the characteristics of crew members living in similar working and living areas and frequent contact. The estimated risk factors for the relatively high incidence of this case can be estimated from the physical structure of the amphibious ship, which cannot be ventilated, and the unavoidable close contact between the crew members who live together for 24 hours. As described above, it is judged that unified mitigation is necessary for ships, such as periodic ventilation, preemptive testing, observing personal quarantine rules, and blocking transmission through prompt contact management in case of a confirmed case, in consideration of the structural characteristics that are vulnerable to the spread of the COVID-19 virus.

## Figures and Tables

**Figure f2-epih-44-e2022065:**
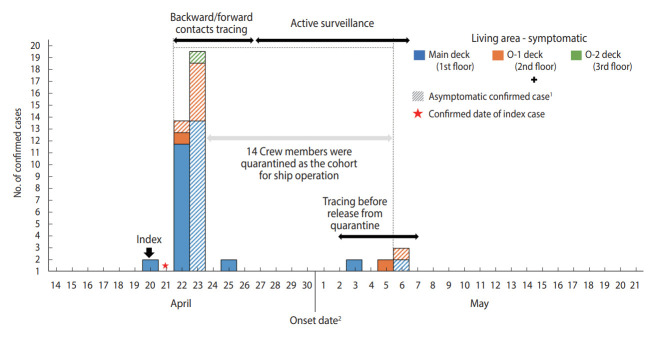


**Table 1.** Demographics of crew members in the Republic of Korea Navy amphibious warfare ship

Characteristics	Cases (n=38)	Non-cases (n=47)	Total (n=85)
Sex			
Male	37 (48.1)	40 (51.9)	77 (100)
Female	1 (12.5)	7 (87.5)	8 (100)
Age (yr)			
19-24	21 (44.7)	26 (55.3)	47 (100)
25-39	7 (38.9)	11 (61.1)	18 (100)
40-49	10 (50.0)	10 (50.0)	20 (100)
Rank			
Seaman - CPO	28 (46.7)	32 (53.3)	60 (100)
Senior & Master CPO	10 (58.8)	7 (41.2)	17 (100)
Officer	0 (0.0)	8 (100)	8 (100)
Working area			
2nd deck (basement)	3 (42.9)	4 (57.1)	7 (100)
Main deck (1st floor)	31 (52.5)	28 (47.5)	59 (100)
O-1 deck (2nd floor)	4 (33.3)	8 (66.7)	12 (100)
O-2 deck (3rd floor)	0 (0.0)	7 (100)	7 (100)
Living area			
Main deck (1st floor)	28 (50.9)	27 (49.1)	55 (100)
O-1 deck (2nd floor)	9 (56.3)	7 (43.8)	16 (100)
O-2 deck (3rd floor)	1 (7.1)	13 (92.9)	14 (100)
Symptoms^1^			
Asymptomatic	22 (57.9)	-	-
Respiratory^2^	12 (31.6)	-	-
Fever	4 (10.5)	-	-
Digestive^3^	2 (5.3)	-	-
Headache & chilling	7 (18.4)	-	-
Anosmia & ageusia	1 (2.6)	-	-
Underlying diseases			
Yes	1 (2.6)	-	-
No	37 (97.4)	-	-
Smoking status			
Non-smoker	35 (92.1)	-	-
Past-smoker	2 (5.3)	-	-
Current-smoker	1 (2.6)	-	-

Values are presented as number (%). CPO, Chief Petty Officer.

1 If a single patient showed several symptoms, it was counted as a duplicate.

2 Includes cough, sputum, and chest discomfort.

3 Includes abdominal pain and diarrhea.

**Table 2.** Results of comparison of infection risk according to demographic characteristics in the Republic of Korea Navy amphibious warfare ship

Characteristics	Total (n=85)	Cases (n=38)	Non-cases (n=47)	Crude^1^	Adjusted^2^
Sex					
Male	77 (100)	37 (48.1)	40 (51.9)	6.48 (0.89, 7.19)	4.52 (0.65, 31.26)
Female	8 (100)	1 (12.5)	7 (87.5)	1.00 (reference)	1.00 (reference)
Age (yr)					
19-24	47 (100)	21 (44.7)	26 (55.3)	1.00 (reference)	1.00 (reference)
25-49	38 (100)	17 (44.7)	21 (55.3)	1.00 (0.36, 1.64)	-^3^
Rank					
Seaman - CPO	60 (100)	28 (46.7)	32 (53.3)	1.31 (0.64, 2.70)	0.91 (0.44, 1.89)
Senior CPO & Officer	25 (100)	10 (40.0)	15 (60.0)	1.00 (reference)	1.00 (reference)
Working area					
2nd deck & main deck (basement & 1st floor)	66 (100)	34 (51.5)	32 (48.5)	3.98 (1.41, 11.23)	3.20 (1.14, 8.99)
O-1 deck & O-2 deck (2nd & 3rd floor)	19 (100)	4 (21.1)	15 (78.9)	1.00 (reference)	1.00 (reference)

Values are presented as number (%) or rate ratio (95% confidence interval). CPO, Chief Petty Officer; COVID-19, coronavirus disease 2019.

1 The crude rate ratio was calculated based on a two-by-two table between COVID-19 infection and other variables.

2 The adjusted rate ratio was calculated using the Mantel–Haenszel method, which is a weighted average of crude rate ratios from the individual stratum-specific two-by-two tables.

3 Due to the high correlation between age and rank, it was excluded from the calculation of adjusted rate ratio.

## SUPPLEMENTARY MATERIALS

Korean version is available at https://www.e-epih.org/.
