# Supplementary material for: Analysis of the on-ship transmission of the COVID-19 mass outbreak on the Republic of Korea Navy amphibious warfare ship
Source: Epidemiol Health. 2022 Aug 11;44:e2022065. doi: 10.4178/epih.e2022065 (PMC9754907; doi:10.4178/epih.e2022065)
Supplement: Supplementary file 1 [file epih-44-e2022065-suppl.docx]

**Title and Author List:**

대한민국 해군 상륙함 코로나19 집단발생사례를 통한 함정내 전파의 특성 분석(Analysis of the on-ship transmission of the COVID-19 mass outbreak on the Republic of Korea Navy amphibious warfare ship)

Soo Hyeon Cho(조수현)^1*^, Young-Man Kim(김영만)^1^, Gyeongyong Seong(성경용)^2^, Sunkyun Park(박선균)^3^, Seoncheol Park(박선철)^4^, Sang-Eun Lee(이상은)^1^ and Young Joon Park(박영준)^1*^

^1^ Central Disease Control Headquarters, Korea Disease Control and Prevention Agency (KDCA), Cheongju, Republic of Korea / 질병관리청 중앙방역대책본부, 충청북도 청주시

^2^ Armed Forces Epidemiological Investigation Center, Seongnam, Republic of Korea / 대한민국 국방부 국군중앙역학조사반, 경기도 성남시

^3^ Office of the Surgeon General, Republic of Korea Navy Headquarters, Gyeryong, Republic of Korea / 대한민국 해군본부 의무실, 충청남도 계룡시

^4^ Department of Information Statistics, Chungbuk National University, Cheongju, Republic of Korea / 충북대학교 정보통계학과, 충청북도 청주시

Corresponding Author: Young Joon Park, MD, MPH Postal address: 28159 Osong Health Technology Administration Complex, 187, Osongsaengmyeong 2-ro, Osong-eup, Heungdeok-gu, Cheongju-si, Chungcheongbuk-do, Korea, E-mail: [pahmun@korea.kr](mailto:pahmun@korea.kr), Tel: 043-719-7950

**ABSTRACT**

Objectives 본 조사는 대한민국 해군 상륙함에서의 코로나19바이러스감염증 집단발생 규모 및 양상, 감염원과 전파경로 등을 규명하기 위해 수행되었다.

Methods 집단발생의 감염원 및 감염경로, 위험요인 등을 조사하기 위해 상륙함의 조사대상 기간 내 전체 승조원 85명을 대상으로 선내 활동 내역, 현장 방문, 인터뷰, 의무기록 추적 등을 조사하였다. 인터뷰의 경우 현장 방문 시 필수 함정 운용 인원14명과 면담을 진행했으며 전체 승조원의 일반적 특성 및 확진자 기초역학조사를 통한 의무기록 추적 등을 실시했다. 조사디자인은 후향적 코호트 조사이며 통계 프로그램을 통해 대상자의 일반적 특성과 감염 유무에 영향을 미칠 것으로 예상되는 변수의 단변량 및 타 변수의 효과가 보정된 다변량 비교위험도를 분석했다.

Results 해군 상륙함에서의 발생률은 44.7%(38명/85명)이다. 주요 전파 경로는 1층 근무자로부터 시작되어 동일 층에 우선 전파되고 이후 다른 층으로 전파된 것으로 추정하였다. 근무 구역의 경우 자연 환기가 가능한 2층 이상 근무자(21.1%)에 비해 환기가 불가능한 1층 이하 근무자(51.5%)의 발생률이 높았으며 발병 위험도 비교 결과 통계적으로 유의했다.

Conclusions 밀집된 환경에서 밀접한 생활을 하는 해군 상륙함의 특성으로 인해 함정내 바이러스 유입 시 발생률이 높은 것으로 추정되므로 향후 함정 내 코로나19를 포함한 다양한 호흡기 감염병의 예방을 위해서는 백신 접종, 주기적 환기, 선제적 검사 등 조치와 함께 확진자 발생 시 신속한 접촉자 관리 등을 통한 전파 차단 등 unified mitigation이 필요할 것으로 판단된다.

**KEYWORDS** COVID-19, Outbreaks, Epidemiology, Military Personnel, Ships

**INTRODUCTION**

2019년 12월 중국 후베이성 우한시에서 원인미상 폐렴 환자의 집단 발생을 시작으로 전세계에 확산된 코로나바이러스감염증19(COVID-19, 코로나19)은 주로 비말을 통한 호흡기전파로 알려져 있으며 바이러스가 포함된 비말이 묻은 매개물(4시간부터 최대 수일 동안 생존) 접촉에 의한 접촉 전파도 가능한 것으로 알려져 있다[1]. 한편 최근에는 밀폐된 제한적 공간에서의 공기 전파도 가능한 것으로 보고되고 있다[2]. 임상증상은 무증상에서 중증까지 다양하게 나타나며, 주요 증상으로는 37.5도 이상의 발열, 인후통, 기침, 호흡곤란 및 오한, 근육통, 두통, 후각 또는 미각소실 등이 있다. ‘20년 1월 코로나19 판데믹 이후, 전세계적으로 교육시설, 종교시설, 목욕탕 및 음식점 등 다중이용시설, 병원 및 사업장 등 다양한 장소와 경로를 통한 집단 사례 발생이 보고되고 있다[3-4]. 코로나19에 의한 다양한 집단 사례 중 선박에서의 코로나19 집단 발병은 일본에서 2020년 2월 중순 탑승자 700여 명이 감염된 D사의 크루즈선 사례가 있으며, 이후 미국 G사의 크루즈선, 브라질의 화물선 등의 사례가 보고되었으며[5-7], 특히 항공 모함을 비롯한 군함에서의 코로나19 집단 사례[8-9]도 보고되어 선박에서의 코로나19 감염 및 전파 위험성이 제기되어왔다. 국내도 ’20년 1월 20일 코로나19 확진자가 최초로 유입된 이후 ‘22년 4월 20일 0시 기준 누적 16,583,220명의 확진자가 발생하고 있으며[10], 우리나라 역시 다양한 집단사례가 지속적으로 발생하고 있으나 사회적 거리두기 및 마스크 착용 등 국민들의 적극적인 개인방역수칙 준수와 방역당국 및 지자체가 공동으로 지역사회에서의 위험집단에 대한 집중관리와 전수조사, 선제검사를 통해 유행이 효과적으로 통제되어 왔다. 특히 집단 생활을 하는 군 부대 인원들은 다양한 감염병에 취약하다고 알려져 있기 때문에[11] 군 조직의 특성상 국방부에서는 보다 강화된 방역 조치가 수행되고 있었다. 그러나 2020년 12월 이후 영국, 남아공, 브라질 유래 주요 변이 바이러스 및 미국 등에서 유래한 기타 변이 바이러스가 국내에서 확인되었으며, 지속적으로 바이러스의 증식과 전파 및 변이 발생이 예상되는 바[12] 집단 발생 사례에 대한 분석과 규명은 상당히 중요하다고 판단된다.

‘21년 4월 23일 국내 해군 상륙함에서의 집단 발생 사례가 인지된 이후, 신속한 역학조사 및 전파 차단을 위해 질병관리청과 국방부와의 합동조사반이 구성되었으며, 본 사례는 국내 해군 상륙함에서의 첫 코로나19 집단 발생 사례로서 코로나19 집단 발병의 규모 및 양상, 감염원과 전파경로 등 역학적 특성을 보고하는 바이다.

**MATERIALS and METHODS**

**Outbreak Recognition**

본 조사는 2021년 4월 20일 모항인 경남 창원시 소재 진해 해군기지를 출항하여 2021년 4월 23일 경기 평택시 소재 평택 해군기지에 정박중인 해군 상륙함의 승조원 중 4월 22일 확진자가 발생하였고 이후 해당 함정 내 승조원 전원(84명)에 대한 전수검사를 진행하여 추가로 32명이 확진되면서 4월 23일 집단 유행을 인지하였다.

**Case Definition and Epidemiologic Investigations**

확진 환자는 상륙함의 조사대상 기간(4월 18일~4월 22일)내 노출이 확인된 사례 중 ‘임상양상에 관계없이 진단을 위한 검사 기준에 따라 상기도에서 채취된 검체에서 RT-PCR법을 사용하여 SARS-CoV-2 바이러스 유전자(E, RdRp 유전자)가 확인된 자’로 정의하였다[13]. 상륙함 승조원 대상 검사는 지표환자의 경우 평택 소재 2차 의료기관에서 실시했으며, 이외 승조원 전원은 현장 검체 채취 후 국군의학연구소에 검사 의뢰되었다. 검체 분석의 경우 국군의학연구소에서 일부 검체를 대상으로 진행되었다. 해군 상륙함 관련 코로나19 집단 발생이 보고된 4월 23일, 질병관리청과 국방부 합동역학조사반이 함내 발생양상, 감염원, 전파경로를 파악하고 추가 전파차단 및 감염관리를 위해 현장역학조사 및 대응조치를 시행하였다. 코로나19 대응지침에 따라 모든 확진자는 원칙적으로 격리 및 치료가 실시되어야 하나[13], 함정의 특성 상 유사시를 대비하기 위해 최소 운용 인원 14명이 함께 선내 코호트 격리 조치를 실시하였다. 따라서 현장 방문 시 개인보호구를 착용하고 코호트 격리 중인 함정 운용 인원들과 면담을 시행할 수 있었다. 별도의 설문 도구를 사용하지는 않았으나, 함정 근무 방식이나 생활 양식 등에 대해 실무자의 의견을 확인하였다. 의무기록의 경우 의무사령부 역학조사관이 확진자 기초역학조사 시 활용한 자체 의무기록을 검토하여 흡연력 및 기저질환 등을 확인하였으며, 해군 역학조사관이 승조원 전수를 대상으로 성별 및 나이 등을 포함한 일반적 특성과 침실 및 근무 구역 등의 정보를 수집하였다. 또한, 유입 경로를 파악하기 위해 확진자의 건강보험심사평가원의 의약품안전사용서비스 내역을 검토하였다. 합동역학조사반은 이와 같이 전체 승조원(85명)을 대상으로 선내 활동 내역, 현장 방문, 면담 및 의무기록 등을 토대로 후향적 코호트조사를 실시하였다.

**Statistical analysis**

R(4.1.2) 버전과 R studio(’21.9) 프로그램 및 R패키지 ‘fmsb’에 구현된 rateratio 함수를 이용하였으며 유의성 기준은 alpha=0.05(95% 신뢰구간)이다. 해당 프로그램을 통해 대상자의 일반적 특성과 COVID-19 감염 유무에 영향을 미칠 것으로 예상되는 변수의 단변량(Crude) 및 타 변수의 효과가 보정된(Adjusted) 비교위험도를 계산해 평가하였다. 이러한 인구학적 특성에 따른 발병위험도(Incidence Rate Ratio, IRR) 비교는 기존 연구[14-17]의 계산식을 참고하였는데, COVID-19 감염 여부와 다른 변수들 사이의 2x2 분할표를 작성한 후 분할표 분석을 시행했다. 해당 분석 시 성별, 나이, 계급 및 근무 구역을 변수로 선택하였다. 다변량 분석의 경우, 타 변수의 효과를 보정하기 위해 추가적으로 stratification 방법인 Mantel-Haenszel 분석법을 활용했다. 이는 다변량 버전의 변수 영향력 계산 시 분할표 자료를 데이터의 범주별로 층화하여 세분화한 후, 각기 다른 층의 rate ratio의 가중 평균으로 타 변수의 효과가 보정된 rate ratio를 제시하는 방법이다. 상대적으로 많지 않은 표본의 수(N=85)를 가진 연구임을 고려하여 비교위험도 평가 시 변수 선택 기준의 경우, 군 부대 특성과 함선의 물리적 구조를 고려하여 범주를 재구성하였다. 나이의 경우 군 계급 조직의 특성을 고려하여 두 그룹(19-24세, 25-49세)으로 나누었으며, 근무 구역의 경우 해군 상륙함의 구조적 특성 상 다중공선성을 고려하여 근무 구역을 자연환기가 불가한 구역(지하~1층)과 자연환기가 가능한 구역(2층~3층)으로 나누었다.

**Ethics statement**

The study was approved by Republic of Korea Armed Forces Medical Command Institutional Review Board (IRB No: AFMC-202202-HR-008-02).

**RESULTS**

해군 상륙함 전체 승조원 85명 가운데 확진자는 38명, 비확진자는 47명으로 발병률은 44.7%이다. 국군의학연구소 보고에 따르면 확진자 중 3명에 대한 S단백질 분석 결과 별도의 주요변이 바이러스는 발견되지 않았다. 지표환자는 30대 남성이자 중사 이하 계급에 속했으며, 4월 21일 자정 경 동거 가족인 자녀의 4월 16일 등원 및 하원을 위해 방문한 어린이집에서 확진자가 다수 발생하고 있다는 소식을 듣고 즉시 함내 격리를 실시했다. 4월 22일 오전 10시 경 평택에 입항하자마자 해당 지역 소재 병원 선별진료소에서 검사를 시행했으며, 당일 오후 5시 양성 판정을 받았다. 지표환자가 진단된 이후 승조원 전수검사를 통해 4월 23일까지 32명의 환자가 추가로 확인되었다. 4월 25일 1명의 유증상 환자가 추가 확진되었으며, 5월 격리 해제 전 검사에서 4명이 추가로 확진되었다(Figure 1). 지표환자의 경우 최초 역학조사 시 무증상이라고 진술하였으나, 의약품안전사용서비스 조사 결과 약국 이용력 및 확진일 약 3주 전부터 기침 증상이 있었음을 확인하였다. 그러나 해당 기간 내 부대 내 확진자 발생이 없었고, 역학적 연관성이 확인되는 이동 동선이 없었으며 지표환자의 동거가족 내 확진자가 발생하지 않은 것을 고려해볼 때, 약 3주 간의 기침 증상은 코로나 바이러스 감염과 큰 관련이 없다고 판단되어 최근 약국 방문력을 기준으로 4월 20일을 증상 발현일로 추정하고 조사 기간을 설정하였다. 따라서 지표환자를 근원환자로 추정하고 4월 18일부터 지표환자가 확진된 22일까지 최소 5일 동안 함내 코로나19 바이러스가 노출된 것으로 추정하였다. 지표 환자는 1층 단독 근무 공간에서 근무하였으나 배의 구조상 화장실, 공용공간 등 함내 여러 장소에서 다른 승조원들과 접촉이 가능했으며, 특히 보급 업무를 담당했던 지표환자의 보직 특성상 불특정 다수와의 밀접한 접촉이 가능했다. 또한 증상발현 약 2일 전으로 추정되는 4월 18일 당직 근무를 실시함으로써 수병들의 점호를 주관하며 1개의 식당에서 당직 근무자들이 함께 식사를 하는 등 기타 승조원들과의 접촉이 평소보다 더 많이 이루어졌다고 볼 수 있었다.

상륙함 승조원의 일반적 특성은 다음과 같다(table 1). 전체 남성 승조원(77명)에서는 48.1%, 여성은 8명 중 단 1명만이 확진 판정을 받았다(12.5%). 연령대 발병률은 40대에서 가장 높은 발병률을 보였으나(50.0%), 군 집단의 특성 상 전체 확진자 중에서는 19-24세에서 높은 발병률(21/38, 55.3%)을 보였다. 계급별로는 중사 이하와 상사 및 원사 계급에서는 발병률이 각각 46.7%와 58.5%였으나, 장교들은 모두 음성이었다. 근무구역에 따라서는 1층에서 근무했던 사람의 발병률이 52.5%로 가장 높았으며, 그 다음으로 지하 1층(42.9%), 2층(33.3%)의 순이었고, 3층에서 근무했던 사람들 중 확진 판정을 받은 사람은 없었다. 마지막으로 생활구역으로 나누었을 때는 1층과 2층에서 생활하던 사람의 발병률이 각각 50.9%, 56.3%로 높았고 3층에서는 총 14명의 거주자 중 1명만이 확진 판정을 받았다(7.1%). 확진자의 임상증상에 있어서는 무증상자가 57.9%(22명), 유증상자는 42.1%(16명)으로, 기침(12명), 두통 및 오한(7명)을 주로 호소하였다. 개인별 특성으로 확진자의 대부분(97.4%)이 비흡연자였다.

인구학적 특성에 따른 발병 위험도는 성별과 연령 및 계급, 근무 구역에 따라 그 결과를 비교하였다(table 2). 다른 변수를 통제하지 않은 단변량 모델의 경우 여성에 비해 남성이(6.48, 95% CI 0.89 - 7.19), 상사 이상에 비해 중사 이하의 계급에서(1.31, 95% CI 0.64 - 2.70) 발병위험도가 높았으나 이는 통계적으로 유의하지 않았다. 근무 구역의 경우 2층과 3층에 비해 지하와 1층에서 근무하는 경우 발병위험도가 높았으며 이는 통계적으로 유의했다(3.98, 95% 1.41 - 11.23). 다른 변수를 통제한 다변량 모델 분석 시에는 군 조직의 특성 상 나이와 계급, 성별 변수 사이의 연관성을 확인하였다. Cramer의 V 통계량을 활용하여 범주형 자료의 상관성을 측정하였는데, 나이와 계급간의 상관계수가 가장 높았다(0.6659). 따라서 다른 변수와의 상관관계가 높았고 확진 유무를 설명하는 데 가장 관련이 적었던 나이 변수를 제외하였다. 다변량 모델의 경우 여성에 비해 남성의 발병위험도가 높았으나 통계적으로 유의하지 않았다(4.52, 95% CI 0.65 - 31.26). 계급의 경우 상사 이상에 비해 중사 이하의 계급에서 발병위험도가 낮았으나 통계적으로 유의하지 않았다(0.91, 95% CI 0.44 - 1.89). 근무 구역의 경우 단변량 모델과 마찬가지로 2층과 3층에 비해 지하와 1층에서 근무하는 경우 발병위험도가 높았으며, 역시 통계적으로 유의한 결과를 보였다(3.20, 95% CI 1.14 - 8.99).

현장조사 및 승조원과의 면담 결과, 식사의 경우 사병식당이 있어 일반 승조원들이 식사하였는데, 평소 배식은 지하와 1층으로 나누었으며 최대한 거리두기를 유지하기 위해 식탁에 아크릴판을 설치하여 한 방향으로 앉아 접촉을 최소화했으나 상륙함의 물리적 공간 특성상 최소 1m 이상 거리두기는 곤란한 것으로 확인되었다. 1층의 경우, 지하 2개 층과 마찬가지로 자연 환기가 어렵고 별도 기계 공조 장치가 있으나 필요시 수동으로 조작하여 운영하고 있었다. 지상 2층에는 상사 및 원사의 생활공간(숙소, 식당), 의무실, 장교식당 및 회의실이 있었으며, 1층 이하의 공간과는 다르게 자연환기가 가능하고 회의실 내 비말 차단을 위한 아크릴판을 설치하는 등 거리두기 유지가 1층 이하의 장소보다 용이했다. 3층은 장교 생활관이었으며, 2층과 마찬가지로 자연환기가 가능하였다. 상륙함 승조원들은 3교대 근무로 함정 정비, 운영, 조리 등 여러 개의 병과 및 승조원이 함께 생활해왔으나 중사 이하의 일반 승조원과 원사 이하의 CPO, 장교 계급으로 이루어진 사관 계급의 생활 공간(침실과 식당, 샤워실과 화장실이 포함)은 철저히 분리되어 있었다. 출항 전 개인 정비를 위해 수병들은 간단한 외출이 허용되며, 하사 이상의 간부 역시 출항 후 필요한 짐을 챙기는 등 개인 시간을 가진 것이 확인되었다. 그러나 코로나19 집단발병을 막기 위해 군내 거리두기는 민간보다 1단계씩 격상되어 2단계로 운영하고 있었으며, 해군 역시 외부인과의 접촉을 최소화하기 위해 불필요한 출타를 줄이고 수병의 경우 휴가 복귀 시 PCR 검사를 필수적으로 시행하고 있었다. PCR 검사에서 음성 판정을 받더라도 14일 간 근무에서 제외하며 출타자끼리 동일 집단 격리함으로써 거리두기를 유지하고 있었다.

**DISCUSSION**

본 조사는 대한민국 해군 상륙함에서의 코로나19바이러스감염증 집단발생 규모 및 양상, 감염원과 전파경로 등을 규명하기 위해 수행되었다. 본 연구의 대상인 해군 상륙함의 전체 발병률은 44.7%이며, 군용 상륙함의 경우 기존 상용되는 화물선이나 관광용 크루즈선과는 다르게 근무자인 승조원 외 다른 탑승객이 없었으므로 지역사회로의 전파는 없었다.

본 사례의 전파경로는 증상이 가장 빠른 근원환자이자 최초 확진자인 지표환자로부터의 타 승조원 전파로 추정된다. 실제로 대부분의 확진자가 지표환자와 같은 중사 이하의 일반 승조원이었으며 상대적으로 접촉이 적은 장교(사관) 계급에서는 확진자가 없었다. 사관 계급이 주로 생활하는 3층 생활구역에서는 단 1명의 확진자가 있었으나, 이는 여군 침실을 이용하던 중사 이하의 여군으로 근무 구역인 1층으로부터 감염되었음을 예상해볼 수 있다. 단, 개별적인 접촉력을 확인할 수 없어 전파 경로를 추정할 때 전파 가능 기간 내 지표환자의 당직 근무 및 평소 보급 업무로 인한 타 계급과의 접촉 가능성도 함께 고려하여야 할 것이다. 근무 공간과 생활 구역이 유사한 상륙함의 구조 상, 1층에서 근무하고 생활하는 지표환자로부터 2층, 3층 승조원으로의 동시 다발적 전파가 있었다고 예상해볼 수 있다. 유입경로의 경우 지표환자보다 증상이 더 빠른 승조원을 발견할 수 없었고, 군 조직 특성 상 외부 출입이 제한되는 점을 고려할 때 본 집단발생사례는 유입 경로 미상의 지표환자로부터 타 승조원이 감염된 것으로 추정해볼 수 있다. 지표환자를 포함하여 의료기관 및 약국 이용자를 분석한 결과 역시 코로나19 유사 증상으로 인한 진료 및 구매 내역 사례를 특정할 수 없었으며, 지표환자 외 민간인 확진자와 접촉점이 있는 역학적 관련성은 확인되지 않았다. 비록 지표환자의 검체 채취 배경에 자녀 등하원시 확진자 교원과의 접촉 가능성이 있었으나, 지표환자의 자녀 및 배우자를 포함한 동거가족 중 추가 확진자가 없어 유입 경로를 확정할 수 없다. 따라서 지표환자의 감염경로를 특정할 수 없으며 지표환자 외 상륙함 내 다른 선행 확진자를 추정하기 어렵다.

발생 규모의 경우 대규모 선박 집단 감염 사례로 평가되는 D사의 선원과 탑승객 전원에 대한 발병률(19.2%), G사의 선원과 탑승객 전원에 대한 발병률(3.4%)을 비교해볼 수 있으며[5-7], 국외 군함 사례의 경우 25% 이상의 발병률을 보인 항공모함 관련 집단 사례[9]와 주요변이 중 하나인 델타형 변이 바이러스가 확인되었음에도 불구하고 승조원 98% 이상의 예방접종 이후 약 6.3%의 발병률을 보인 군함 사례[8]가 있다. 해당 시기별 바이러스 유형과 예방접종 여부 등에 따른 차이는 있겠으나, 해당 집단은 주요 변이 바이러스가 확인되지 않았다. 바이러스 유형 역시 별도의 유전형 검사는 시행되지 않았으나 2021년 4월 말 당시 국내 확진자 대부분의 유전형이 GH clade인 점을 고려하였을 때[12] 관련 특이 사항은 없는 것으로 추정된다. 국내 사례로는 2021년 7월 승조원의 예방접종 시행 없이 해외 파병 임무를 수행했던 구축함에서 약 90.4%의 발병률을 보인 집단 발생 사례가 있었는데[18], 해당 사례는 관련 검사에서 델타형 변이바이러스가 확인되었다. 본 사례는 비교적 높은 전파력을 보이는 주요 변이 바이러스[19]에 해당하지 않으나 위와 같이 국내외 사례와 비교했을 때 유사 집단 사례보다 다소 높은 발병률로 해석된다. 이처럼 높은 발병률의 원인은 다음과 같이 추정해볼 수 있다. 첫째, 해당 함선은 취역한지 약 30년이 되어 1층 이하 내부 환기가 제한되는 동시에 비말 확산에 취약하였다. 함내 특정 장소는 자연환기가 불가하였으며, 기계 환기 장치의 주기적 가동이 어려워 질병관리청의 환기 가이드라인[13]을 준수하기에 적절하지 않았다. 따라서 자연 환기가 불가한 1층 이하 생활 및 활동 공간 내 체류나 접촉을 집단 감염의 주요 위험요인으로 추정하였다. 본 연구의 분석 결과 역시 근무지 기준 2층 이상은 자연환기가 되므로 전파 위험률이 1층 이하보다 낮았으며, 이는 단변량 및 다변량 분석을 통해 통계적으로 유의함을 확인하였다. 따라서 자연 환기가 불가능한 구역에서는 공조시설을 수시로 가동하여 주기적인 환기를 실시해야 할 것이다. 둘째, 본 사례의 계급 간 전파 위험 요인으로 예상해볼 수 있었던 당직 근무자들의 공동 식당 이용을 고려할 수 있다. 마스크 착용이 불가한 함내 식당이 코로나19 전파 장소가 될 수 있다[20]는 기존 연구 결과에 따라 식사 시에도 강화된 거리두기 유지가 중요한 것으로 판단되므로[21-22] 접촉 최소화를 위해 배식 시간을 순차적으로 실시하며, 식사 간 대화금지를 재차 교육해야 할 것이다. 셋째, 해당 상륙함의 확진자 절반 이상이 무증상이었던 점을 고려할 때 군은 기존에 알려진 무증상 감염자의 전파 가능 사실과 최대 잠복기를 염두하여[23] 휴가 복귀자에 대한 증상 감시를 실시 해야하며 승선 및 출항 전 유증상자를 파악하고, 함내 PCR 검사가 불가능한 상황을 고려하여 출항 전 모든 승조원에 대해 선제적 검사를 실시해야 할 것이다. 마스크 착용 등 예방수칙 준수와 함께 출항 후 유증상자 발견 시 즉각 생활공간을 격리하며 신속한 접촉자 관리를 하는 등 전파 차단에 초점을 맞추어 추가 감염을 최소화하는 것이 요구된다. 마지막으로 본 집단발생 사례가 코로나19 예방접종이 도입되기 전 발생한 점을 고려해야할 것이다. 비슷한 함내 환경에서 방역 수칙을 준수하는 동시에 대부분의 승조원들이 예방접종을 마친 경우, 코로나19 발병률을 줄인다는 최근의 연구 결과[8]를 볼 때 출항 전 충분히 예방접종을 완료해야 할 것이다.

한편, 본 조사는 다음과 같은 제한점이 있다. 첫째, 군사 보안의 특성상 함정 내 CCTV등을 활용한 승조원들의 이동 패턴 파악이 불가했고, 진술에만 의존하여 객관성 확보에 한계가 있었으며 배의 구조를 정확히 설명하기 위한 도면 및 설계도 확보 역시 불가능했다. 둘째, 외부인 출입 제한으로 공기동역학 실험을 할 수 없었기 때문에 공동 식당을 비롯한 함내 공기 유동의 특성을 파악하지 못했다. 마지막으로 이번 집단 사례 발생 당시, 승조원 예방접종이 추진되지 않아 예방접종력과의 연관성 및 예방접종의 효과를 분석하지 못했다.

본 조사는 대한민국 해군 상륙함에서 발생한 최초의 코로나19 집단 발생 현황을 분석하여 집단 발생 규모 및 양상, 전파경로 등을 규명했다. 유입 경로 미상의 승조원으로부터 시작된 감염으로, 근무 구역과 생활 구역이 유사하고 접촉이 잦은 승조원 생활의 특성상 동시 다발적 전파가 있었음을 예상할 수 있다. 본 사례의 비교적 높은 발병률의 추정 위험요인은 자연환기가 불가한 상륙함의 물리적 구조와 24시간 공동 생활을 하는 승조원 간의 불가피한 밀집·밀접 접촉 등을 추정해볼 수 있다. 이와 같이 함정은 코로나19 바이러스 확산에 취약한 구조적 특성을 고려하여 주기적 환기, 선제적 검사, 개인방역수칙 준수와 더불어 확진자 발생 시 신속한 접촉자 관리 등을 통한 전파 차단 등 unified mitigation이 필요할 것으로 판단된다.

**REFERENCES**

[1] Centers for Disease Control and Prevention. COVID-19: How COVID-19 Spreads; 2021-7-14 [cited 2022 Jun 2]. Available from: https://www.cdc.gov/coronavirus/2019-ncov/prevent-getting-sick/how-covid-spreads.html.

[2] Zahra Noorimotlagh, Neemat Jaafarzadeh, Susana Silva Martínez, et al. A systematic review of possible airborne transmission of the COVID-19 virus (SARS-CoV-2) in the indoor air environment. Environmental Research 2021;193:e110612.

[3] Park SY, Kim YM, Yi S, Lee S, Na BJ, Kim CB, et al. Coronavirus disease outbreak in call center, South Korea. Emerg Infect Dis 2020;26(8):1666-1670.

[4] Kang YJ. Characteristics of the COVID-19 outbreak in Korea from the mass infection perspective. J Prev Med Public Health 2020;53:168-170.

[5] Moriarty LF, Plucinski MM, Marston BJ, et al. Public Health Responses to COVID-19 Outbreaks on Cruise Ships - Worldwide, February-March 2020. MMWR Morb Mortal Wkly Rep 2020;69:347-352.

[6] Maeda H, Sando E, Toizumi M, et al. Epidemiology of Coronavirus Disease Outbreak among Crewmembers on Cruise Ship, Nagasaki City, Japan, April 2020. Emerg Infect Dis 2021;27(9):2251-2260.

[7] Hatzianastasiou S, Mouchtouri VA, Pavli A, et al. COVID-19 Outbreak on a Passenger Ship and Assessment of Response Measures, Greece, 2020. Emerg Infect Dis 2021;27(7):1927-1930.

[8] Servies TE, Larsen EC, Lindsay RC, et al. Notes from the Field: Outbreak of COVID-19 Among a Highly Vaccinated Population Aboard a U.S. Navy Ship After a Port Visit — Reykjavik, Iceland, July 2021. MMWR Morb Mortal Wkly Rep 2022;71:279-281.

[9] Kasper MR, Geibe JR, Sears CL, et al. An outbreak of Covid-19 on an aircraft carrier. N Engl J Med 2020;383:2417-26.

[10] Korea Disease Control and Prevention Agency(KDCA). COVID-19 Vaccination and Domestic Occurences(Regular Briefing); 2022-4-20 [cited 2022 Apr 25]. Available from: https://www.kdca.go.kr/board/board.es?mid=a20501020000&bid=0015&list_no=719335&cg_code=C01&act=view&nPage=1.hwp.

[11] Yoo H, Gu SH, Jugn J, Song DH, Yoon C, Hong DJ, et al. Febrile respiratory illness associated with human adenovirus type 55 in South Korea military, 2014-2016. Emerg Infect Dis 2017;23:1016-1020.

[12] Kim Il-Hwan, Park Ae kyung, Kim Jeong-Min, Kim Heui Man, Lee Nam-Joo, Kim Eun-Jin et al. COVID-19 Variant Surveillance Study in the Republic of Korea. Public Health Weekly Report, PHWR 2021;14(13):724-733.

[13] Korea Disease Control and Prevention Agency(KDCA). COVID-19 response guidelines for local government(10th); 2021 [cited 2022 Apr 1]. Available from: https://www.kdca.go.kr/board/board.es?mid=a20507020000&bid=0019.hwp.

[14] Yunyun Jiang, Andrew B. Lawson, Li zhu, Eric J. Feuer. Interval Estimation for Age-Adjusted Rate Ratios Using Bayesian Convolution Model. Frontiers in Public Health 2019;7:2296-2565.

[15] Isabel dos Santos Sliva. International Agency for Research on Cancer. Cancer Epidemiology: Principles and Methods - Chapter 14. Dealing with confounding in the anlaysis; 1999, p. 305-331.

[16] R. Vijh, C.H. Ng, M. Shirmaleki et al. Factors associated with transmission of COVID-19 in long-term care facility outbreaks. Journal of Hospital Infection 2022;119:118-125.

[17] Ray M. Merrill. Introduction to Epidemiology. 7^th^ ed. Burlington: Jones & Bartlett Learning Co.; 2017. P. 151-153.

[18] Korea Disease Control and Prevention Agency(KDCA). Result of Epidemiological Investigation of COVID-19 cluster at Somali Sea Escort Task Group; 2021 Aug 10 [cited 2022 Jun 2]. Available from:https://www.kdca.go.kr/board/board.es?mid=a20501010000&bid=0015&list_no=716455&cg_code=&act=view&nPage=1.

[19] Choi JY, Smith DM. SARS-CoV-2 Variants of Concern. Yonsei Med J 2021;62(11):961-968.

[20] Kakimoto K, Kamiya H, Yamagishi T, Matsui T, Suzuki M, Wakita T. Initial investigation of transmission of COVID-19 among crew members during quarantine of a cruise ship—Yokohama, Japan, February 2020. MMWR Morb Mortal Wkly Rep 2020;69:312-3.

[21] European Union Health Gateways. Advice for ship operators for preparedness and response to the outbreak of COVID-19; 2020. Available from: https://www.healthygateways.eu/Portals/0/plcdocs/EU_HEALTHY_GATEWAYS_COVID-19_MARITIME_20_2_2020_FINAL.pdf?ver=2020-02-21-123842-480.pdf.

[22] World Health Organization. Interim guidance for operational considerations for managing COVID-19 cases/outbreak on board ships; 2020 [cited 2022 Apr 1]. Available from: https://www.who.int/publications/i/item/operational-considerations-for-managing-covid-19-cases-outbreak-on-board-ships.

[23] Gao W, Lv J, Pang Y et al. Role of asymptomatic and pre-symptomatic infections in covid-19 pandemic. BMJ 2021;375:n2342.
